# Additive Effect of Platelet Rich Fibrin with Coronally Advanced Flap Procedure in Root Coverage of Miller’s Class I and II Recession Defects—A PRISMA Compliant Systematic Review and Meta-Analysis

**DOI:** 10.3390/ma13194314

**Published:** 2020-09-27

**Authors:** Saurav Panda, Anurag Satpathy, Abhaya Chandra Das, Manoj Kumar, Lora Mishra, Swati Gupta, Gunjan Srivastava, Monika Lukomska-Szymanska, Silvio Taschieri, Massimo Del Fabbro

**Affiliations:** 1 Department of Periodontics and Oral Implantology, Siksha ‘O’ Anusandhan University, Bhubaneswar 751003, India; sauravpanda@soa.ac.in (S.P.); anuragsatpathy@soa.ac.in (A.S.); abhayadas@soa.ac.in (A.C.D.); manojkumar@soa.ac.in (M.K.); 2 Department of Biomedical, Surgical and Dental Sciences, Università degli Studi di Milano, 20122 Milano, Italy; silvio.taschieri@unimi.it; 3 Department of Conservative Dentistry and Endodontics, Institute of Dental Sciences, Siksha ‘O’ Anusandhan University, Bhubaneswar 751003, India; loramishra@soa.ac.in; 4 Private Practice, Gainesville, FL 32603, USA; swati.gup9@gmail.com; 5 Department of Prosthodontics, Institute of Dental Sciences, Siksha ‘O’ Anusandhan University, Bhubaneswar 751003, India; drgunjans22@gmail.com; 6 Department of General Dentistry, Medical University of Lodz, 92-213 Lodz, Poland; monika.lukomska-szymanska@umed.lodz.pl; 7 Dental Clinic, IRCCS Istituto Ortopedico Galeazzi, 20161 Milano, Italy

**Keywords:** gingival recession, periodontal disease, periodontal regeneration, blood platelet

## Abstract

Aim: This systematic review and meta-analysis aims to assess the additive effect of leukocyte and platelet-rich fibrin (L-PRF) on coronally advanced flap (CAF) procedures in root coverage of Miller’s class I and II gingival recession defects. Review methodology: A comprehensive search in MEDLINE (PubMed), Scopus and CENTRAL (the Cochrane Central Register of Controlled Trials), along with an additional hand search, provided eight randomized clinical trials to be included in this review. A total of 167 patients with 470 gingival recession defects were analyzed. A meta-analysis was carried out to assess the change in gingival thickness (GT), width of keratinized gingiva (WKG), root coverage percentage (%RC), clinical attachment level (CAL) and recession depth (RD) at all follow-ups between CAF alone and CAF + L-PRF groups for all included studies. A subgroup analysis was carried out based on recession type (single/multiple). Results: Overall, a significant improvement in GT, CAL and RD was found when treated with CAF + L-PRF. There was a trend for a positive effect in terms of an increase in WKG when using L-PRF, especially in the treatment of single recession, though significance was not achieved (*p* = 0.08 overall). The results of heterogeneity among the subgroups were varied and were found to be greater than 91.3% for GT and 32.8% for WKG. Conclusion: L-PRF when used in addition to CAF showed favorable results for the treatment of class I and II gingival recession defects.

## 1. Introduction

Gingival recession is the downward migration of attachment apparatus leading to an apical displacement of gingival margin and oral exposure of the root [1]. The most common patient-related symptoms include compromised aesthetics and lingering hypersensitivity [2]. Hence, the root coverage procedures aim primarily at reducing hypersensitivity and improving the gingival aesthetics. The other objectives include increasing the width of keratinized gingiva, improving gingival biotype, preventing root caries, managing noncarious cervical lesions (NCCL) and maintaining tooth-supporting structures. Various root coverage procedures and techniques are employed based on numerous factors, such as recession type, location, adequacy of keratinized gingiva, single or multiple teeth involved, gingival biotype, presence of NCCL and need of a secondary donor site [3].

Aesthetic concern is one of the main reasons behind the treatment of gingival recession defects. So far, mean root coverage achieved with a variety of periodontal plastic procedures has been reported to be 70–80% [4]. It has been well accepted that one of the main prerequisites to achieve a satisfactory outcome in root coverage procedures is adequate blood supply. Another important aspect to be considered for achieving excellent root coverage is the type of recession defect. Numerous classifications have been proposed to identify, diagnose and determine the prognosis of the recession defects, among which Miller’s classification of gingival recession is the most accepted and widely used one [5]. According to Miller, the prognosis is considered good with root coverage percentage up to 100% in cases of class I and II gingival recession, while treating single recession defect types in mandibular anterior teeth with the augmentation of free gingival grafts.

One of the most reliable techniques, with the best long-term and stable clinical results in the management of gingival recession, is the coronally advanced flap (CAF) procedure, provided there are adequate attached gingiva [6]. This procedure is most beneficial for the treatment of multiple adjacent recession type defects, however, it can also be employed to treat single recession defects. This procedure has been used independently, but has also been used in addition with connective tissue grafts [7], barrier membranes [8], acellular dermal matrices [9], amniotic chorion membranes [10], enamel matrix derivatives and platelet concentrates [11,12], which has shown advantageous clinical outcomes.

According to a recent systematic review, owing to its predictable outcomes, connective tissue graft procedures (CTG), in combination with coronally advanced flaps, are still considered the gold standard for root coverage [13]. Nevertheless, this procedure comes with its own limitations of second site morbidity and patient discomfort. In order to avoid this, clinicians have been using biomaterials such as acellular dermal matrix derivatives or platelet rich preparations as an alternative to CTG, in combination with less invasive techniques such as pedicle or coronally advanced flaps [14,15,16].

The introduction of platelet-rich preparations has revolutionized the concept of healing and regenerative dentistry [17,18]. Platelets secrete a significant amount of growth factors such as vascular endothelial growth factors, platelet-derived growth factors and cytokines, as well as many other adhesion factors. Leukocytes, one of the major components in platelet concentrates, play a substantial role in wound healing. These cells have immune regulatory, as well as anti-infectious, properties [19,20]. Leukocyte and platelet-rich fibrin (L-PRF) is considered to be a second-generation platelet concentrate produced as a natural concentrate without any anticoagulants or gelling agents [21]. L-PRF consists of a tetra-molecular fibrin entity with cytokines, platelets and stem cells embedded in it. L-PRF amounts for 95% of cellular concentration of platelets and more than 50% of leukocytes, distributed in this three-dimensional tetra-molecular fibrin network. The first few millimeters of the clot beyond the red cell base holds the largest proportions of platelets, and is regarded as the most regenerative. The concentration is found less in the part away from the red cell base [17]. The L-PRF membrane allows the sustained release of growth factors like platelet-derived growth factors, insulin-like growth factors, vascular endothelial growth factors, basic fibroblast growth factors etc., which are involved in cell proliferation and migration [22]. L-PRF also plays an important role as a biodegradable scaffold, favoring development of microvascularization and enabling it to guide epithelial cell migration on its surface [23].

There is a lot of clinical evidence supporting the applications of L-PRF in soft tissue healing, in particular, for the coverage of Miller’s class I and II gingival recession defects when combined with various root coverage procedures [24,25]. A few studies were also conducted in combination with coronally advanced flap, assessing the comparative efficacy with the gold standard [7,26,27].

A systematic review [28] assessed the adjunctive effect of PRF on various root coverage procedures after carrying out a meta-analysis with only three studies comparing CAF + L-PRF versus CAF alone, and did not find any statistically significant differences. However, L-PRF was found to have beneficial effects for recorded clinical parameters. Since the comparison of CAF + L-PRF versus CAF + CTG was not significant, L-PRF was suggested as a substitute to CTG which has the potential to show a beneficial outcome on bone and keratinized tissue healing, as well as postoperative discomfort reduction. Recently, Li et al. [29] evaluated the effect of autologous platelet concentrates when used with CAF. They included 5 studies to assess the effect of L-PRF and showed L-PRF could exert an additive effect to CAF. However, the systematic review did not assess the variation or modification of the CAF procedure, preparation protocol for L-PRF and type of recession defects (single/multiple).

With respect to the addition of newer evidences, and taking into account the effect of the above-mentioned variability, the objective of this review was to assess the additive effect of L-PRF on the CAF procedure in the management of class I and II gingival recession defects.

## 2. Experimental Section

This review strictly adheres to Preferred Reporting Items for Systematic Reviews and Meta-Analyses (PRISMA) guidelines. The protocol of this review was registered at PROSPERO with registration number CRD42019126012.

### 2.1. Research Question

Does L-PRF have any additive effect when used in combination with CAF in the management of class I and II gingival recession defects?

P Participants with class I and II gingival recession defects
I Intervention with L-PRF with CAF
C Comparison with CAF alone
O Outcome variable, such as gingival thickness (GT), width of keratinized gingiva (WKG), % root coverage (%RC), clinical attachment level (CAL), recession depth (RD) or complete root coverage (CRC).

### 2.2. Search Methods

Databases such as MEDLINE (PubMed), SCOPUS, CENTRAL (the Cochrane Central Register of Controlled Trials), EMBASE (Excerpta Medica dataBASE) and WoS (Web of Science) were searched in a systematic manner with relevant keywords and strategically employing ‘AND’, ‘OR’ and ‘NOT’.

The detailed search string read as follows: “((((Root Coverage) OR Gingival Recession) OR Coronally Advanced Flap Procedure)) AND (((Leukocyte-Platelet rich fibrin) OR L-PRF) OR Platelet Concentrate)”. The search was last performed on 15th February 2020. In addition, a manual search was also carried out in the recent issues of dental-related journals: *Clinical Oral Investigations*, *European Journal of Oral Sciences*, *Journal of Periodontics and Restorative Dentistry*, *Journal of Dental Research*, *Journal of Dentistry*, *Journal of Periodontal Research*, *Journal of Clinical Periodontology*, *Journal of Periodontal and Implant Science and Journal of Periodontology*. The bibliography column of relevant clinical reports and reviews were screened for any additional eligible clinical studies.

### 2.3. Selection Criteria

The following criteria were used for the selection of potentially eligible studies from the list of studies identified through the electronic search and additional hand search:

- Parallel or split-mouth randomized controlled trials (RCTs);
- Studies employing L-PRF in adjunct to CAF as test group comparing to use of CAF alone as control group for root coverage;
- Studies treating maxillary/mandibular class I or II gingival recession defects.
- Studies with participants free of any systemic diseases nor taking medications that could potentially influence the outcome of periodontal therapy;
- Studies with follow-up periods of 6 months.

### 2.4. Selection of Studies

The studies retrieved from the electronic database searches were compiled into a citation manager software (EndNote v7.0, Clarivate Analytics, New York, NY, USA) to remove the duplicates. After the removal of duplicate items, all the studies were screened based on titles and abstracts by two independent reviewers (MDF, SP). The potentially eligible studies were subjected to a full text assessment and tagged under included studies if they were found to satisfy the selection criteria. A third reviewer, an expert in the field (ST), was consulted to resolve any disagreements that arose between the first two reviewers. The excluded studies were listed with the reason for their exclusion.

### 2.5. Data Extraction

The data collection was made using an Excel spreadsheet (2010 version, Microsoft, Redmond, WA, USA) to retrieve relevant detailed information from the included studies for qualitative synthesis. The data extraction from all included studies was carried out separately by three independent reviewers (ACD, SG, MK) in order to eliminate errors in the extraction of variables and outcomes. If any of the included studies were reported missing, incomplete or had unclear information, the authors were contacted over telephone or email to enquire for the details of missing or unclear data.

The below-mentioned data were retrieved from individual studies:

- Demographic data of participants
- Type of gingival recession (multiple or single)
- Type of RCT and number of patients
- Type of equipment and protocol used for preparation of L-PRF
- Follow-up period
- Study sponsor and its setting
- Outcome variables, relative to baseline and postoperative recession defect characteristics e.g.: gingival thickness (GT), width of keratinized gingiva (WKG), % root coverage (%RC), clinical attachment level (CAL), recession depth (RD) and complete root coverage (CRC).

### 2.6. Data Synthesis

The extracted data were subjected to both qualitative and quantitative analysis. The qualitative data and the demographics from all of the included studies were tabulated, seen in Table 1. The quantitative data extracted for different outcomes were constructed and subjected to meta-analysis in case of the availability of at least two studies with similar outcome measurements and comparable follow-up periods. Mean differences (MD) with 95% confidence intervals (CI) were used to synthesize the data for the continuous primary outcomes, such as gingival thickness (GT), width of keratinized gingiva (WKG), root coverage percentage (%RC), clinical attachment level (CAL) and recession depth (RD). The statistical unit of measurement for the analysis of each outcome was the tooth site instead of the patient.

The quantitative data were analyzed using Review Manager 5.3 software (RevMan 5.3, Version 5.3.5, Copenhagen, Denmark: The Nordic Cochrane Centre, The Cochrane Collaboration, 2014.). A fixed-effects model for analysis was employed for heterogeneity *i^2^ < 60%, p > 0.05*. When the heterogeneity *i^2^ > 60%, p < 0.05*, a random-effects model was used for meta-analysis.

### 2.7. Risk of Bias Analysis

The analysis for the included studies was performed using the Cochrane risk of bias tool (RevMan 5.3, Version 5.3.5, Copenhagen, Denmark: The Nordic Cochrane Centre, The Cochrane Collaboration, 2014.) for randomized controlled trials. The included studies were assessed based on: Random sequence generation, allocation concealment, blinding of participants and personnel, blinding of outcome assessments, incomplete outcome data and selective reporting. These domains were graded as high, unclear or low risk based on individual assessments. Two independent reviewers (LM, GS) carried out the assessment for all of the included studies. In the case of any disagreements, a third reviewer (AS) was consulted to reach consensus. The included studies were categorized as low, if all of the aforementioned criteria were at low risk, except if one or less of the domains were at unclear risk, and the studies were regarded as high, if one or more criteria were at high risk. A medium risk was allotted to the studies if two or more criteria were unclear.

## 3. Results

**Figure 1:**
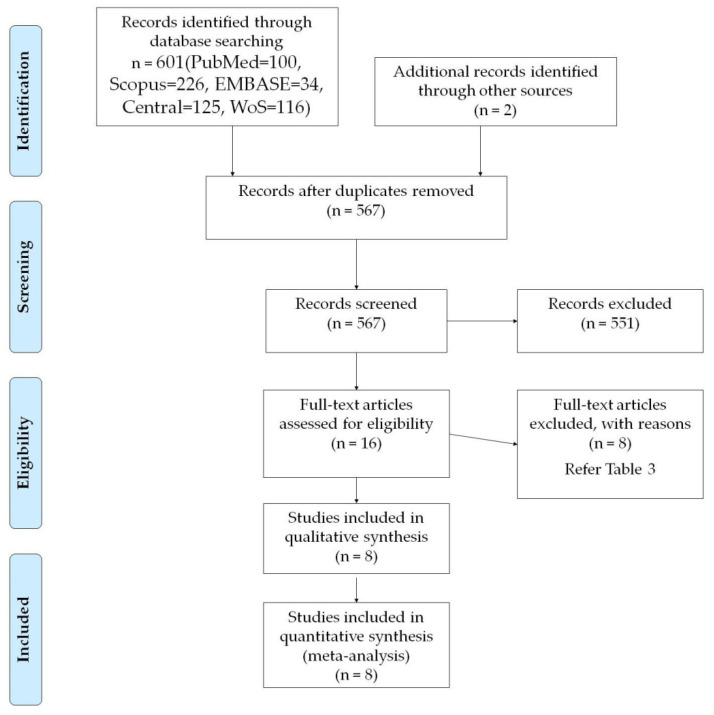
PRISMA—flow chart for study selection.

A comprehensive search in electronic databases, along with the additional hand search, provided 8 RCTs to be included in this systematic review. The systematic flow chart for the study identification process is detailed in Figure 1.

A total of 8 RCTs selected for this systematic review compared the additive effect of L-PRF with the CAF procedure in the management of class I and II gingival recession. Out of the 8 RCTs, 4 studies [30,31,32,33] had a split–mouth design and the other 4 studies [34,35,36,37] had a parallel design. Similarly, 4 RCTs [31,34,35,36] treated single recessions, and the remaining 4 studies [30,32,33,37] treated multiple adjacent recessions. The included trials had heterogeneity based on the study design and recession type. The heterogeneity was addressed by carrying out a subgroup analysis based on the recession type. The surgical protocol employed among all included trials was similar in carrying out the CAF procedure, except 1 study [24] employed a modified approach (MCAF) and another study [31] used orthodontic buttons (CAF-B) to secure the suture knots and attain postoperative stability. The CAF procedure was employed by providing horizontal and vertical incisions to release flap tension and was carried out by elevating a muco-periosteal or split thickness flap under local anesthesia.

A total of 167 subjects with 470 gingival recession defects among the included trials were evaluated in this systematic review. All the studies were analyzed both qualitatively and quantitatively. The general characteristics of all included trials are provided in Table 1 and Table 2. The excluded studies with valid reasons of exclusion are provided in Table 3.

### 3.1. Meta-Analysis

The quantitative analysis was made by carrying out a meta-analysis for comparison between the CAF + L-PRF group and the CAF alone group. The change in GT, WKG, %RC, CAL and RD at all follow-ups were compared between both of the groups for all included studies.

#### 3.1.1. Change in Gingival Thickness (GT)

**Figure 2:**
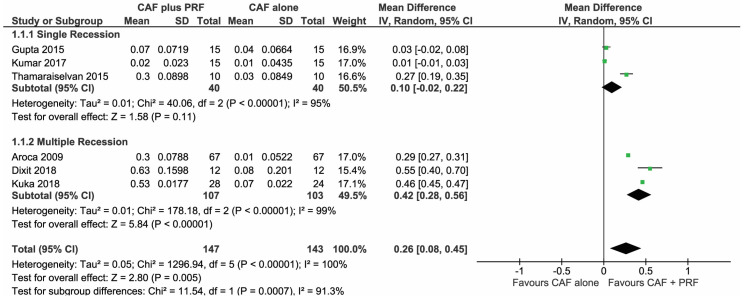
Comparison of change in gingival thickness (GT) between coronally advanced flap procedures (CAF) + L-PRF versus CAF alone with 6–12 months follow-up.

A total of 6 studies [30,32,34,35,36,37] were analyzed in the forest plot to find the change in GT to be significantly higher for CAF + L-PRF groups than the CAF alone group, with a mean difference of MD 0.26 (95% CI (0.08,0.45), *p* = 0.005) post 6–12 months follow-up, as seen in Figure 2.

Studies [30,32,37] that treated multiple adjacent recessions with CAF + L-PRF showed more favorable outcomes than CAF alone (MD 0.42mm, *p* < 0.0001), however, the change in GT could not be significantly expressed in studies [34,35,36] treating single recessions with similar techniques (MD 0.10mm, *p* = 0.11).

#### 3.1.2. Change in Width of Keratinized Gingiva (WKG)

**Figure 3:**
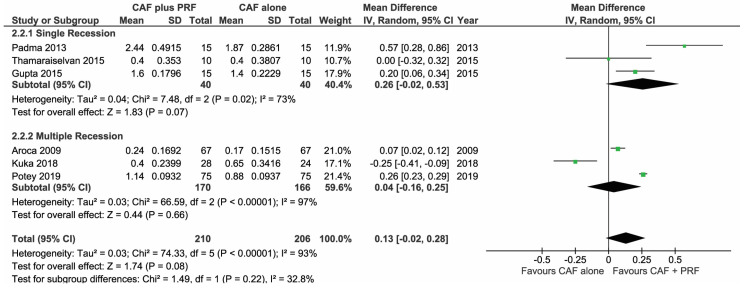
Comparison of change in width of keratinized gingiva (WKG) between CAF + L-PRF versus CAF alone with 6–12 months follow-up.

A total of 6 studies [30,31,33,34,35,37] were analyzed in the forest plot to find the change in WKG among the CAF + L-PRF group and the CAF alone group, suggesting no significant mean difference (MD 0.13 95% CI (−0.02, 0.28), *p* = 0.08) post 6–12 months follow-up, seen in Figure 3.

The subgroup analysis revealed no significant difference. The adjunctive use of L-PRF with CAF did not show any significant favorable change in WKG while treating single, as well as multiple, adjacent recessions.

#### 3.1.3. Change in Root Coverage Percentage (%RC)

**Figure 4:**
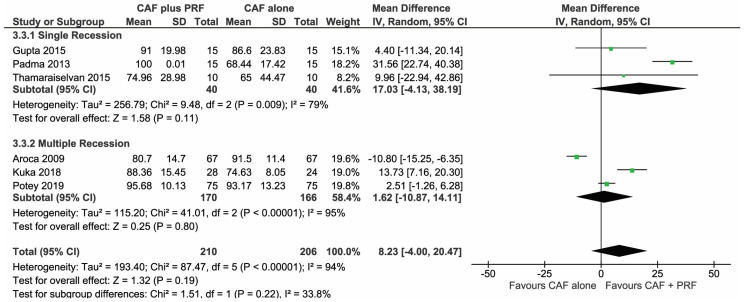
Comparison of change in root coverage percentage (%RC) between CAF + L-PRF versus CAF alone with 6–12 months follow-up.

A total of 6 studies [30,31,33,34,35,37], which were analyzed in the forest plot to find the change in %RC among the CAF + L-PRF group and the CAF alone group, showed no significant mean difference (MD 8.23 95% CI (−4.00,20.47), *p* = 0.19) post 6–12 months follow-up, seen in Figure 4.

#### 3.1.4. Change in Clinical Attachment Level (CAL)

**Figure 5:**
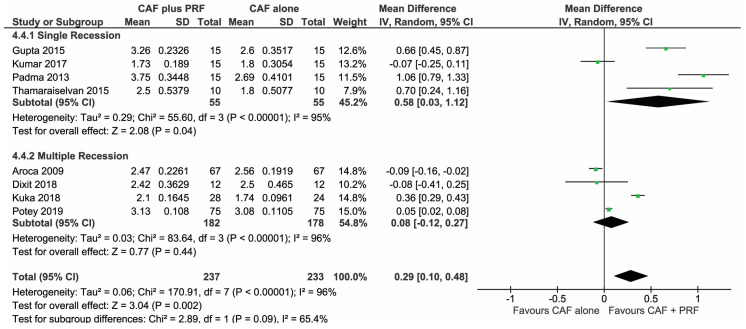
Comparison of change in clinical attachment level (CAL) between CAF + L-PRF versus CAF alone with 6–12 months follow-up.

All 8 studies [30,31,32,33,34,35,36,37] were analyzed in the forest plot to find the change in CAL to be significantly higher for CAF + L-PRF groups compared to the CAF alone group, with a mean difference of MD 0.29 (95% CI (0.10,0.48), *p* = 0.002) post 6–12 months follow-up, seen in Figure 5.

Studies [31,34,35,36] that treated single gingival recessions with CAF + L-PRF showed more favorable outcomes than CAF alone (MD 0.58, *p* = 0.04), however, the change in CAL could not be significantly expressed in studies [30,32,33,37] treating multiple adjacent recessions with similar techniques (MD 0.08, *p* = 0.44).

#### 3.1.5. Change in Recession Depth (RD)

**Figure 6:**
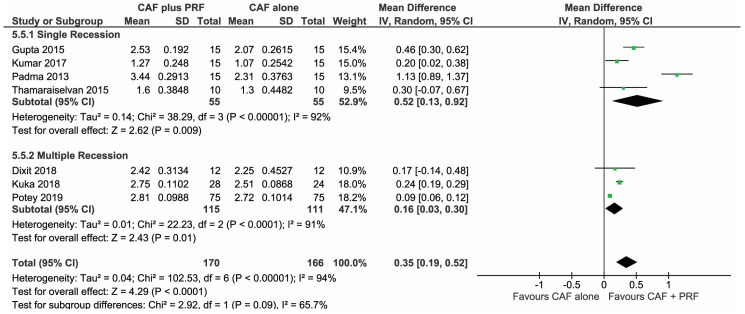
Comparison of change in recession depth (RD) between CAF + L-PRF versus CAF alone with 6–12 months follow-up.

A total of 7 studies [31,32,33,34,35,36,37], which were analyzed in the forest plot to find the change in RD among the CAF + L-PRF group and the CAF alone group, showed an overall significant mean difference (MD 0.35, 95% CI (0.19,0.52), *p* < 0.0001) post 6–12 months follow-up, seen in Figure 6.

### 3.2. Effect of Preparation Protocol for L-PRF

The included studies reported using two different protocols of preparation for L-PRF. A total of 6 studies [30,31,33,35,36,37] followed the Choukroun’s protocol [17] of centrifuging whole blood at 3000 rpm for 10 min, while the other 2 studies [32,34] followed protocol suggested by Pinto and Quirynen [38] of centrifuging at 2700 rpm for 12 min. In total, 5 studies [31,32,33,35,36] did not report the specifications or make/brand of centrifuge used.

### 3.3. Effect of Inclusion of Smokers

In total, 1 study [30] clearly mentioned the inclusion of smokers as participants. The results of the study were quite intriguing as the complete root coverage (CRC) observed in the group treated with CAF alone (50 out of 67 defects) was found to be higher than the group treated with CAF + L-PRF (35 out of 67 defects). This adverse result could be relatable to the smokers included in the study.

### 3.4. Effect of Modifications in CAF Procedure

Out of the eight included studies, 2 studies [30,33] used a modified approach for the CAF procedure. In total, 1 study [33] used orthodontic buttons for securing the sutures for postoperative stability and reduced interdental plaque accumulation. This study showed the highest CRC among the included studies (62 out of 75 defects) for the group treated with L-PRF along with this modified approach.

Contrary to this, another study [30], which employed Zuchelli’s modification of providing a submarginal incision at interdental areas and raising a split-full-split flap to coronally advance the flap with horizontal suspended sutures around contact points, found a higher CRC in the group treated with the modified CAF alone.

### 3.5. Risk of Bias Summary

**Figure 7:**
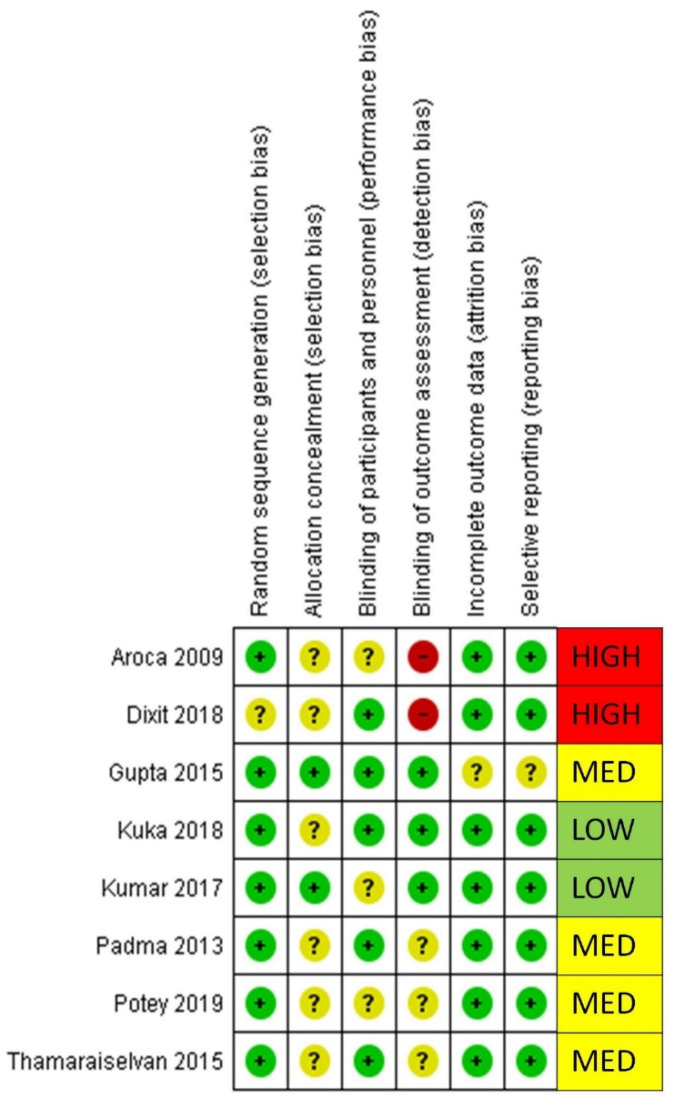
Risk of bias summary: review authors’ judgments about each risk of bias item for each included study.

The risk of bias summary is provided based on the authors’ judgement for all of the included studies, seen in Figure 7. All of the included studies were at low to medium risk, except 2 studies [30,32] were found to have a high risk of bias due to their inability to blind outcome assessors.

## 4. Discussion

In recent years, many studies have claimed that platelet concentrate may improve soft tissue healing, stabilize the initial clot and induce flap revascularization in root coverage procedures. L-PRF generally showed a positive effect in cases with Miller’s class I and II gingival recession defects. The regenerative potential of L-PRF is harnessed when used in surgical treatment along with CAF. The present systematic review and meta-analysis assessed the additive effect of L-PRF when used as an adjunct to CAF for management of class I and II gingival recession defects. In spite of the high heterogeneity among the included studies, the overall effect showed a favorable result with improvement in the clinical parameters when treated with L-PRF along with CAF. This review also compared the change in clinical parameters viz. GT, WKG, %RC, CAL and RD among CAF alone and CAF + L-PRF groups.

Overall, a significant improvement in GT, CAL and RD was found when treated with CAF + L-PRF. This result can be attributed to the additive regenerative potential of L-PRF [39,40]. L-PRF has a positive influence on cell proliferation, migration, adhesion, differentiation and inflammation, which proves its therapeutic potential in regenerative approaches [41]. An in vitro study [42] showed that “L-PRF stimulated cell proliferation of osteoblasts (135% of the control), periodontal ligament cells (130% of the control), and gingival fibroblasts (120% of the control) during a 3-day culture period (all *p* < 0.05).” Therefore, L-PRF modulates the proliferation of cells in a specific manner, which may be beneficial for periodontal regeneration.

A subgroup analysis was carried out to address the possible differences among the outcomes of the studies regarding the selection of recession type (single or multiple adjacent recessions). This aspect has not been considered in the previous systematic reviews [28,29]. The results of heterogeneity among the subgroups were varied and were found to be greater than 91.3% for GT and 32.8% for WKG. This attribute can be a result of variation in the patients’ selection criteria of studies, inclusion of patients with confounding factors like smoking habits, the study design of trials (parallel/split-mouth), the preparation protocol of L-PRF, or the use of modified flap designs of CAF. In general, single recessions received a greater benefit from the adjunctive use of L-PRF compared to multiple recessions. In three parameters (GT, WKG, %RC), the advantage did not achieve significance, but was very close and with a clear trend in favor of the L-PRF + CAF group. In multiple recession cases, only GT and RD had a significant benefit of L-PRF, while in other parameters the difference was clearly nonsignificant.

However, improvement in GT seems to be greater in studies treating multiple adjacent recessions, rather than in treatment of single recessions. This may be because of the availability of adequate flap coverage due to the availability of wide attached gingiva [14]. The advantages of treating multiple adjacent recession type defects with CAF include improved pedicled blood supply, secured repositioned flap against multiple teeth and surgical accessibility for periosteal scoring to release flap tension [14]. In respect to flap tension, it has been suggested that the flap tension is inversely related to the recession reduction [43].

Root coverage percentage (%RC) did not show any significant results due to variation across the studies. Among the selected studies, only 1 study [30], which included smokers, did not provide a positive result favoring the CAF + L-PRF group. This may have been due to the detrimental effect of smoking on the peripheral blood cells within the L-PRF, leading to ambiguous results [44]. None of the studies reported about the root surface conditions (i.e., presence of caries, abrasions or restorations). According to Pini-Prato et al. [45], “the presence of NCCL and loss of interdental tissues adversely affect the complete root coverage following surgical procedures.” Since smoking and root surface condition are important predictors of surgical outcomes of root coverage procedures, it should be carefully examined clinically and discussed with patients prior to the surgical procedure. This could also have contributed to the varied success in root coverage among the included studies.

Increasing the width of keratinized tissue to an adequate amount, as well as reducing probing depth, may lead to a favorable prognosis on periodontal tissue health and prevent soft tissue recession. As platelet concentrates have shown favorable effects on keratinocytes proliferation in several in vitro studies [46,47], it is reasonable to assume that they may be beneficial for the stimulation of keratinized tissue formation. According to the results of the present review, there was a trend for a positive effect in terms of an increase in WKG when using L-PRF, especially in the treatment of single recession, though significance was not achieved (*p* = 0.08 overall). Only 1 study [37] reported a better result for the group treated with CAF alone. This might be due to a long-term follow-up of 12 months in this study. The study reports an increase in gingival thickness with L-PRF but failed to demonstrate an additional benefit with other parameters at the end of 12 months follow-up.

CRC is an important criterion for evaluating the success of any root coverage therapy. The postoperative location of the displaced flap in the CAF procedure determines its success. According to a study by Pini-Prato et al. [48], “postoperative location of the gingival margin (GM1) seems to affect the probability of complete root coverage: the more coronal the gingival margin after suturing, the greater the probability of achieving CRC.” The study also proposed that a marked coronal displacement of the gingival margin (GM1 > 2 mm) while suturing would result in 100% root coverage. However, none of the studies for this systemic review reported the degree of coronal flap displacement carried out postoperatively. For this parameter, there seems to not be a clear advantage of adding L-PRF to CAF procedures, and a high heterogeneity in outcomes was found in both single and multiple recession subgroups. It is suggested that future studies evaluating root coverage will take into account the level of coronal flap displacement, as said above.

It is also important to note that the autologous blood spin in each centrifuge, owing to the differences in rotor size, angulation of tubes, composition of tubes and vibration, will result in different biological PRF clots. We observe that the included studies were not homogenous in the type of equipment used for centrifugation. Moreover, the speed and duration of the centrifugation varied among the studies. It is concluded that L-PRF clots fabricated at lower centrifugation speeds and times improve growth factor release and cellular behavior, owing to higher cellular content and growth factor accumulation. This has now been shown in many studies on L-PRF published since 2014 [49].

In our analysis, there was evidence for an improvement in recession depth change when L-PRF was used for both single and multiple recession. Though heterogeneity among studies was detected, all studies showed a trend for greater improvement of recession depth in the L-PRF group. Differences in the mean reduction of recession depth can be explained based on the selection of the size of the defect sites. As put forward by Pini-Prato et al. [50], the sites with deeper recession defects tend to respond more favorably than shallower sites. The same serves as the limitation of this systematic review. A subgroup analysis based on recession depth and recession width could provide us with a detailed insight.

The beneficial effect of L-PRF along with CAF is perhaps owed to its mechanical and biological properties which aid in periodontal healing. L-PRF is used as a membrane under the CAF, which is basically obtained on the compression of a clot. The compression of an L-PRF clot improves the tensile strength, modulus of elasticity and toughness, which provides a better clinical handling [51]. Slow polymerization in the L-PRF preparation protocol also delays the release of growth factors over a period of 7 days, providing benefits of faster healing and reduced postoperative discomfort [52]. The L-PRF membrane also has been found to exhibit antibacterial effects on most of periodontopathogens like *Prevotella intermedia*, *Fusobacterium nucleatum* and *Aggregatibacter actinomycetemcomitans*, as well as exhibit especially strong inhibitory effects on *Porphyromonas gingivalis* [53].

## 5. Conclusions

In general, a favorable effect on parameters related to soft tissue healing was observed when L-PRF was used in addition to CAF for treatment of class I and II gingival recession defects. Nevertheless, heterogeneity among studies might account for some discrepancies observed. Furthermore, standardization of the preparation protocol is important to achieve an optimal effect of L-PRF in regenerative procedures. In addition to favorable biological effects, L-PRF can be considered for its low cost and ease of preparation, as reported by most studies.

## Figures and Tables

**Table 1:** General characteristics of included studies.

Author & Year	Recession Type	No. of Patients	Smoker	Defects (Test/ Control)	Age Range	Male/ Female	Follow-up (Months)	Complete Root Coverage (CRC)	
								Test	Control
Aroca et al. 2009 [24]	Multiple	20	Yes	67/67	22–47	5/5	1, 3 & 6	35/67	50/67
Padma et al. 2012 [25]	Single	15	No	15/15	18–35	NR	1, 3 & 6	15/15	0/15
Gupta et al. 2015 [26]	Single	26	No	15/15	20–50	16/10	3 & 6	12/15	11/15
Thamaraiselvan et al. 2015 [27]	Single	20	No	10/10	21–47	18/2	6	5/10	5/10
Kumar A et al. 2017 [28]	Single	36	No	15/15	25–45	34/2	3 & 6	9/15	4/15
Dixit N et al. 2018 [29]	Multiple	12	No	12/12	18–50	7/5	3 & 6	NR	NR
Kuka S et al. 2018 [30]	Multiple	24	No	28/24	21–41	11/13	12	15/28	8/24
Potey et al. 2019 [31]	Multiple	20	No	75/75	22–47	16/4	3 & 6	62/75	59/75

**Table 2:** Preparation protocol of leukocyte and platelet-rich fibrin (L-PRF) among included studies.

Author and Year	Study Design	Intervention Type		PRF Preparation Protocol		
		Test	Control	Equipment	Speed (RPM)	Time (min)
Aroca et al. 2009 [24]	Split Mouth	MCAF with L-PRF	MCAF	EBA 20 Centrifuge, Hettich, Tuttlingen, Germany	3000	10
Padma et al. 2012 [25]	Split Mouth	CAF with L-PRF	CAF	Not Mentioned	3000	10
Gupta et al. 2015 [26]	Parallel	CAF with L-PRF	CAF	REMI centrifuge, REMI Labs, India	2700	12
Thamaraiselvan et al. 2015 [27]	Parallel	CAF with L-PRF	CAF	Not Mentioned	3000	10
Kumar A et al. 2017 [28]	Parallel	CAF with L-PRF	CAF	Not Mentioned	3000	10
Dixit N et al. 2018 [29]	Split-mouth	CAF with L-PRF	CAF	Not Mentioned	2700	12
Kuka S et al. 2018 [30]	Parallel	CAF with L-PRF	CAF	EBA 20 centrifuge,	3000	10
				Hettich, Tutlingen, Germany		
Potey et al. 2019 [31]	Split Mouth	CAFB with L-PRF	CAFB	Not Mentioned	3000	10

**Table 3:** List of excluded studies.

Author and Year	Reason for Exclusion
Jankovic et al. 2010 [32]	Comparison with Enamel Matrix Derivative
Jankovic et al. 2012 [33]	Comparison with Connective Tissue Graft
Patel et al. 2018 [34]	Comparison between microsurgical and conventional technique
Rehan et al. 2018 [35]	Comparison with amniotic membrane
Ramireddy et al. 2018 [36]	Treatment of recession by glass ionomer cement and L-PRF
Brignardello 2018 [37]	Uncertain Randomization and Blinding
Culhauglu et al. 2018 [38]	Dose dependant L-PRF vs. Connective Tissue Graft
